# Enhancing visibility and hemostasis during necrosectomy for walled-off necrosis: the “dual-gel method”

**DOI:** 10.1055/a-2344-8503

**Published:** 2024-07-08

**Authors:** Kazuki Hama, Haruka Toyonaga, Tatsuya Ishii, Masayo Motoya, Toshifumi Kin, Tsuyoshi Hayashi, Akio Katanuma

**Affiliations:** 1 37009 Center for Gastroenterology, Teine Keijinkai Hospital, Sapporo, Japan

Endoscopic necrosectomy is an effective treatment for walled-off necrosis (WON); however, bleeding complications can be life threatening and require an immediate response 1 . Blood accumulation within the WON compartment makes identifying bleeding blood vessels difficult. Furthermore, the use of clips for hemostasis increases the risk of leaving long-term remains within the WON. Recently, a method for maintaining visibility during gastrointestinal bleeding involving the injection of a gel with an appropriate viscosity (Viscoclear; Otsuka Pharmaceutical Factory, Inc., Tokushima, Japan) was reported 2 3 . Additionally, the hemostatic effect of a self-assembling peptide gel (PuraStat; 3-D Matrix Europe SAS, Lyon, France) used during gastrointestinal endoscopic procedures has been reported 4 5 . Purastat is an aqueous peptide solution that becomes neutral upon contact with blood and body fluids, and its peptide molecules form fibers in solution to form a peptide hydrogel. This hydrogel quickly coats the bleeding point and stops the bleeding. In this case, we successfully employed a novel “dual-gel method” that uses Viscoclear and PuraStat to achieve hemostasis during endoscopic necrosectomy.

A 58-year-old man presented with significant exudative bleeding during endoscopic necrosectomy. The bleeding continued unabated, and the exact location of the bleeding point was unclear because blood had pooled within the WON ( Fig. 1 ). An auxiliary injection cap (BioShield irrigator; US Endoscopy, Mentor, Ohio, USA) was used to free the channel, and Viscoclear was injected via an injection cap ( Fig. 2 ). The injection of Viscoclear facilitated the separation of blood and gel, thereby enabling identification of the bleeding point ( Fig. 3 ). Subsequently, PuraStat injections resulted in effective hemostasis ( Fig. 4 ). The gel-forming properties of PuraStat repaired the injured vessel wall, ensured stability, and sustained its hemostatic effect ( Fig. 5 ).

**Fig. 1:**
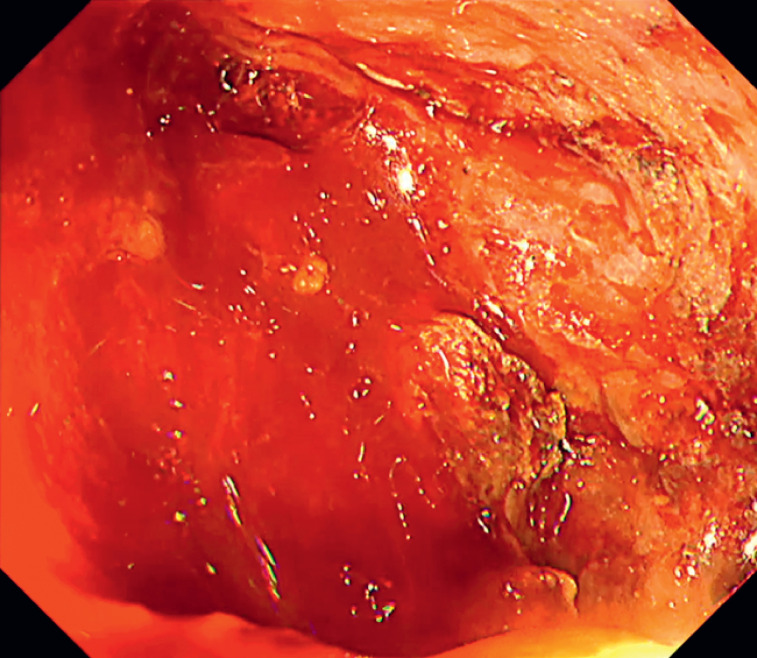
The bleeding persisted, and the source of the bleeding remained uncertain due to the accumulation of blood in the walled-off necrosis.

**Fig. 2:**
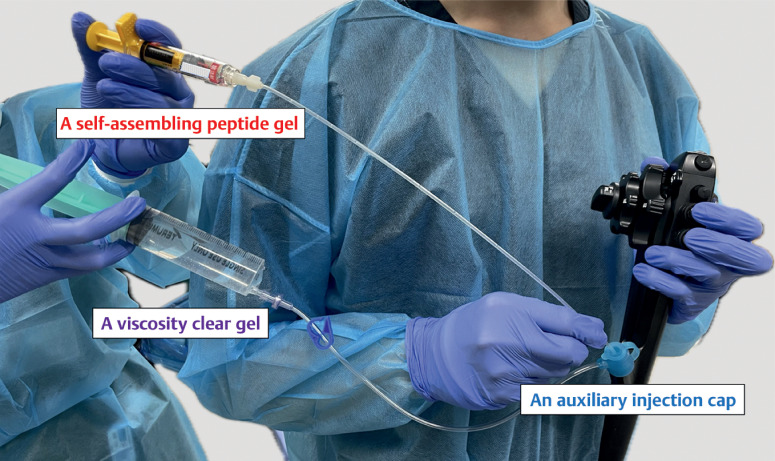
An auxiliary injection cap (BioShield irrigator; US Endoscopy, Mentor, Ohio, USA) was used to free the channel, and a viscous clear gel (Viscoclear; Otsuka Pharmaceutical Factory, Inc., Tokushima, Japan) was injected via the irrigation line. Subsequently, a self-assembling peptide gel (PuraStat; 3-D Matrix Europe SAS, Lyon, France) was injected through the forceps channel.

**Fig. 3:**
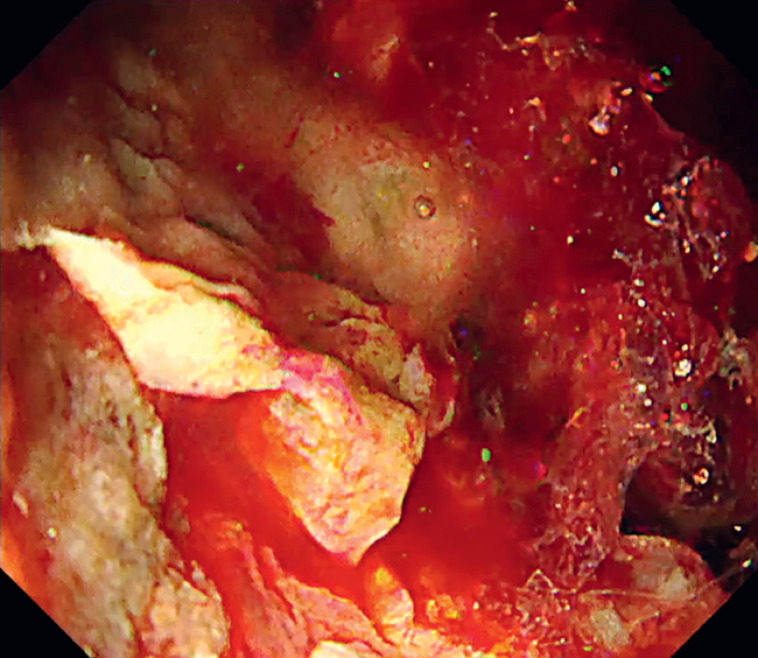
The injection of Viscoclear (Otsuka Pharmaceutical Factory, Inc., Tokushima, Japan) facilitated the separation of blood and gel, thereby enabling identification of the bleeding point.

**Fig. 4:**
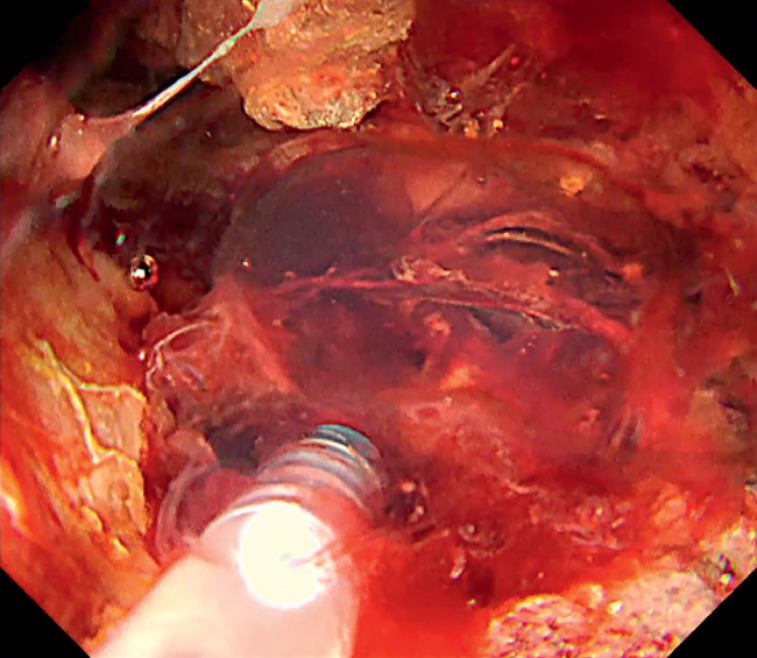
Effective hemostasis was achieved following injection of PuraStat (3-D Matrix Europe SAS, Lyon, France).

**Fig. 5:**
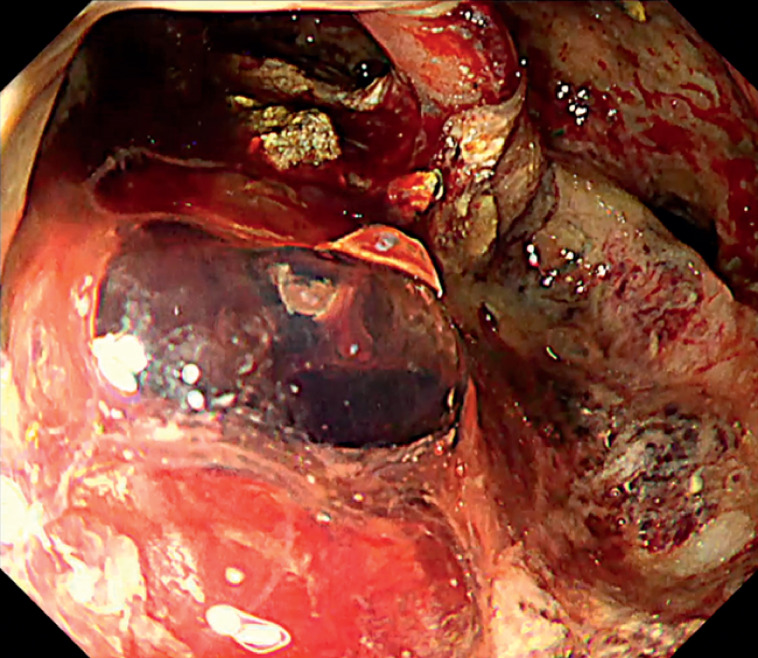
The gel-forming property of PuraStat (3-D Matrix Europe SAS, Lyon, France) ensured its stability in the walled-off necrosis, sustaining its hemostatic efficacy.

The “dual-gel method” is a useful hemostatic technique for managing exudative bleeding within confined spaces of WON ( Video 1 ).

Endoscopic hemostasis using the “dual-gel method” for bleeding during endoscopic necrosectomy.Video 1

Endoscopy_UCTN_Code_TTT_1AS_2AJ

## Notes

Endoscopy E-Videos https://eref.thieme.de/e-videos
*E-Videos* is an open access online section of the journal *Endoscopy* , reporting on interesting cases and new techniques in gastroenterological endoscopy. All papers include a high-quality video and are published with a Creative Commons CC-BY license. Endoscopy E-Videos qualify for HINARI discounts and waivers and eligibility is automatically checked during the submission process. We grant 100% waivers to articles whose corresponding authors are based in Group A countries and 50% waivers to those who are based in Group B countries as classified by Research4Life (see: https://www.research4life.org/access/eligibility/ ). This section has its own submission website at https://mc.manuscriptcentral.com/e-videos .
